# Incidence of peripheral arterial disease in the ARTPER population cohort after 5 years of follow-up

**DOI:** 10.1186/s12872-015-0170-6

**Published:** 2016-01-12

**Authors:** Ma. Teresa Alzamora, Rosa Forés, Guillem Pera, José Miguel Baena-Díez, Antonio Heras, Marta Sorribes, Marta Valverde, Laura Muñoz, Xavier Mundet, Pere Torán

**Affiliations:** Primary Healthcare Centre Riu Nord-Riu Sud, Institut Català de la Salut, Santa Coloma de Gramenet, Barcelona, Spain; Research Unit Barcelonès Nord Maresme, ICS-IDIAP Jordi Gol, Barcelona, Spain; Department of Medicine, Universitat Autònoma de Barcelona, Bellaterra, Spain; Primary Healthcare Centre La Marina, Institut Català de la Salut, Barcelona, Spain; Primary Healthcare Centre Numància, Institut Català de la Salut, Barcelona, Spain; Research Unit Barcelona, ICS-IDIAP Jordi Gol, Barcelona, Spain

**Keywords:** Peripheral arterial disease, Incidence, Cardiovascular risk factors

## Abstract

**Background:**

To know the epidemiology (prevalence, incidence, progression and morbidity and mortality associated) of peripheral artery disease in general population and the factors associated with this progression is essential to know the evolution of atherosclerosis and develop preventive strategies. The aim of the study was to determine the incidence of PAD after 5 years of follow-up population-based cohort ARTPER, and the evolution of Ankle brachial Index (ABI) in this period.

**Methods:**

Peripheral artery disease incidence analysis after 5 years of follow-up of 3786 subjects > 50 years old. Peripheral artery disease incident when the second cross section Ankle brachial Index was <0.9 in any of the lower limbs, with normal baseline (0.9 to 1.4).

**Results:**

Between 2012 and 2013 2762 individuals (77 % participation) were re-examined . Finally analyzed 2256 subjects (after excluding pathological Ankle brachial Index) followed for 4.9 years (range 3.8 to 5.8 years), totalling 11,106 person-years. Peripheral artery disease 95 new cases were detected, representing an incidence of 4.3 % at 5 years and 8.6 per 1000 person-years (95 % CI 6.9 to 10.5) being higher in men (10.2, 95 % CI 7.4 to 13.5) than in women (7.5, 95 % CI 5.5 to 9.9). Linear correlation between the baseline Ankle brachial Index and the second cross section was low (*r* = 0.23).

**Conclusions:**

The incidence of peripheral artery disease in ARTPER cohort was 8.6 cases per 1000 person-years, being higher in men, especially <65 years. The correlation between two measures Ankle brachial Index after 5 years of follow-up was low. One might consider whether Ankle brachial Index repeated measures could improve the correlation.

## Background

Atherosclerosis is currently considered to be a systemic, chronic and progressive illness, having complex pathogenic mechanisms. It occurs in coronary heart disease, cerebrovascular disease, erectile dysfunction and peripheral artery disease (PAD).

The prevalence of PAD of the lower extremities, particularly in the asymptomatic forms, is high. [1] Globally speaking, 202 million people have PAD. In Europe, there has been an increased prevalence of this disease by 13.8 % over the past decade [1]. In countries having low cardiovascular risk such as Spain, the prevalence is situated between 3.7 and 7.6 % [2–5].

PAD is a major cause of decreased quality of life, lower life expectancy and is a major cause of morbidity and mortality [6–10]. With regard to quality of life, 10–20 % of the subjects with PAD have intermittent claudication [6, 7] and up to 50 % may have atypical symptoms in the lower extremities [7]. In addition, PAD triples the risk of mortality and major cardiovascular events including myocardial infarction and stroke [8–10].

Several population-based studies have evaluated the association between the incidence of PAD and the risk factors associated with the progression of this disease [11, 12]. However, the incidence of PAD in Spain has only been studied in selected populations and not in the general population [13, 14]. As for the progression of PAD, there is little agreement among the different studies published to date. Some authors consider the progression of the disease to be demonstrated by a change in ABI from normal to pathological (<0.9 or ≥1.4) [15, 16]; while other authors consider a specific decrease in ABI values [1, 16–18] and still others consider the progression of PAD to occur only when there are vascular events. As for the evolution of ABI values over time, while some authors have described decreases in said values [19–22] while other researchers have reported increases in these values [15, 17, 18].

A decrease in ABI values along follow-up period has also been associated with an increase in both cardiovascular morbidity and mortality from other causes (cardiovascular and not cardiovascular) [17].

In subjects with high vascular risk, the determination of ABI may provide relevant information on the presence of sub-clinical arteriosclerosis and future vascular events. From the perspective of primary prevention, the development of strategies to allow for the identification of sub-clinical atheromatous is needed. One strategy to achieve this may be to detect PAD in the lower extremities using the ABI. REASON is a validated pre-screening test used in our country to determine asymptomatic candidates based on ABI [23].

It is fundamental to understand the epidemiology of PAD in the general Spanish population as well as the factors associated with its progression in order to evaluate the evolution of arteriosclerosis and develop preventative strategies. The main objective of this study was to determine the incidence of PAD after 5 years of follow-up of the ARTPER cohort, as well as the evolution of ABI over the same study period.

## Methods

ARTPER is a population cohort of 3786 subjects over the age of 50, recruited between 2006 and 2008 from 24 Primary Health Centres of the metropolitan Barcelona area and Barcelonés Nord-Maresme (approximately 600,000 inhabitants). Follow-up was conducted via telephone and clinical history reviews were made every 6 months from their creation until 2012 (date when the cohort was re-examined in situ). A description of the study methodology was previously published [4, 24].

Two health care professionals trained in the procedure performed the ABI measurements in a standardized manner [6]. An ABI < 0.9 was considered to be indicative of PAD, while an ABI ≥ 1.4 represents arterial calcification.

### Definition of peripheral artery disease incidence

A decrease in abnormal ABI values (<0.9) in either of the two lower extremities in the second ABI determination at 5 years compared to normal baseline ABI (from 0.9 to 1.4) was considered to indicative a PAD event.

### Progression of the illness

The progression of PAD is considered on finding a decrease >10 % in the minimum ABI values of either of the extremities over time compared to baseline.

### Statistical methods

All of the variables were subject to a thorough quality control process. Continuous variables are described as mean and standard deviation while the categorical variables are expressed as frequency and percentage. Comparisons of continual variables were made with the Student’s t tests and categorical variables were compared with the Chi-square tests. The following dependent variables were studied as well as their association with other variables: 1.- incidence of PAD, using Cox regression models to determine the association with potentially associated factors, adjusted by age, gender and baseline ABI; 2.- disease progression, using logistic regression models to determine the association with potentially associated factors, adjusted by age and gender. In all cases, the possibility of creating transformations or clusters to improve the fit of the model was analysed. For the two dependent variables, a multivariate model was developed which initially included all of the variables with *p* < 0.2 in the bivariate models adjusted by age and gender (and baseline ABI in the Cox models). The models having the best fit to the data were determined using the Akaike criterion, residual behaviour, and Harrell’s C index and proportionality (Cox models) and Hosmer-Lemeshow (logistics models), considering their interpretability and biological plausibility. Some variables that were significant (*p* < 0.05) in the bivariate models were no longer significant when adjusted for by others. In the final multivariate models, only variables with *p* < 0.05 were included. In the Cox models, the baseline ABI was adjusted for, even though this is not revealed in the tables. All comparisons were bilateral and the confidence interval was 95 %. The Stata 13 statistical package was used.

### Ethics

This study was approved by the Ethical Committee of Primary Health Care. All participants signed informed consent to participate.

## Results

**Fig. 1:**
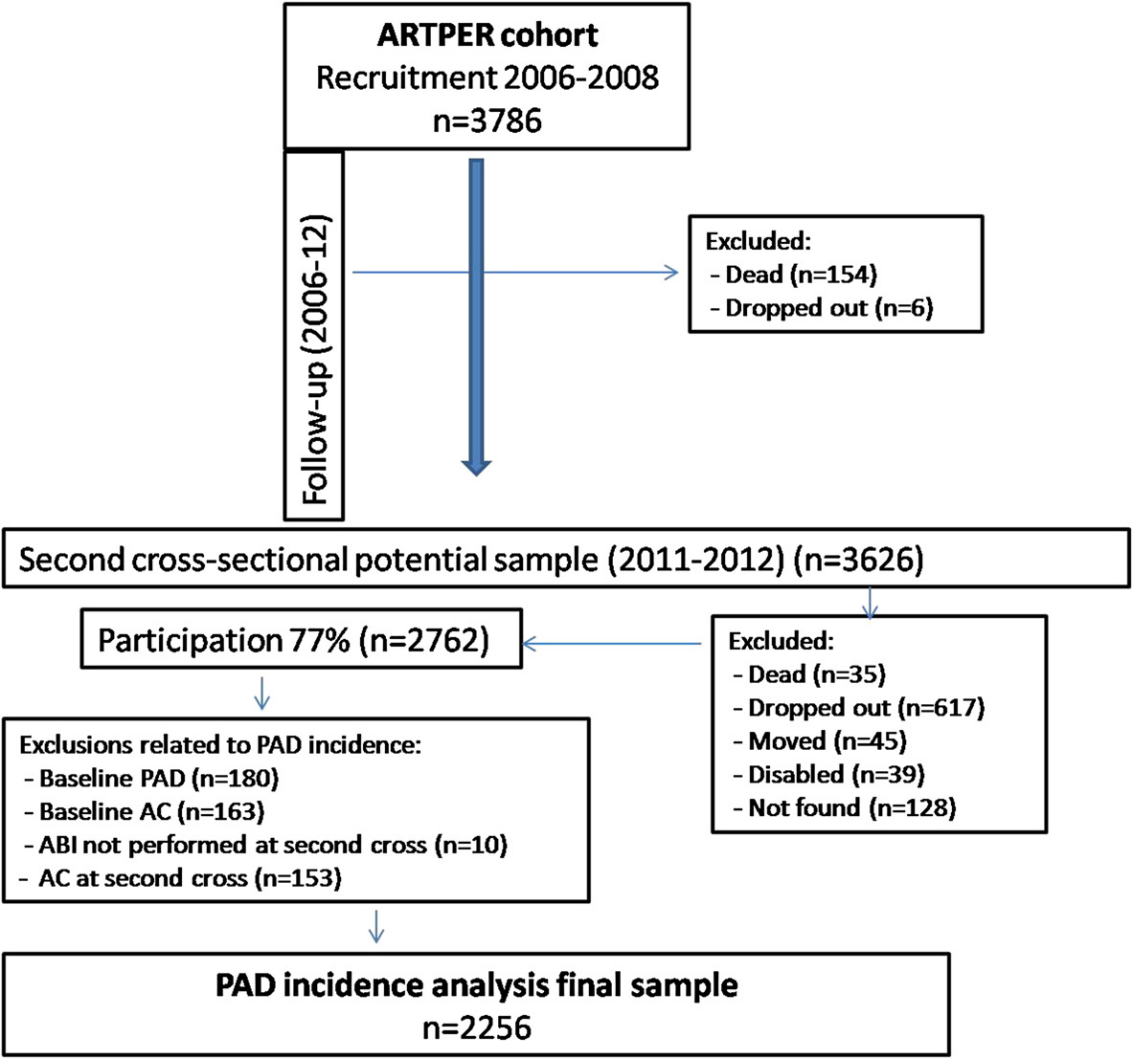
Flow Chart ARTPER cohort

A total of 3786 patients >55 years of age were recruited from March 2011 to September of 2012. Of these 2762 individuals were evaluated in the second ABI measurements (77 %). On comparing the study participants (*n* = 2762) with the non-participants (*n* = 1024) several differences were found with regard to age (64 vs 68 years, *p* < 0.001), female gender (55 % vs 51 %, *p* = 0.013), smoking habit (56 % non-smokers vs 52 %, *p* = 0.025), physical exercise (5734 METS in 14 days vs 4743, *p* < 0.001), hypertension (54 % vs 53 %, *p* < 0.001), hypercholesterolemia (49 % vs 45 %, *p* = 0.009), diabetes (15 % vs 19 %, *p* = 0.009), cardiovascular risk (REGICOR 5.8 % vs 6.6 %, *p* < 0.001). No differences were found in relation to the body mass index (29 in both groups) and clinical history of intermittent claudication (10 % in both groups). The incidence of PAD was analysed in 2256 subjects with normal ABI were included (0.90– 1.40). Patients with a baseline ABI <0.9 (*n* = 180) and ≥ 1.40 (*n* = 163) and those showing calcification in the follow-up (*n* = 153) or who were unable to undergo the second ABI measurement (*n* = 10) were excluded (Fig. 1). The baseline characteristics of the 2256 subjects analysed are shown in Table 1. The study participants were followed for 4.9 years (range: 3.8–5.8 years), with a total of 11,106 individuals-year. The mean baseline ABI was 1.093, being 1.099 at follow-up. Ninety-five new cases of PAD were identified, representing an incidence of PAD of 4.3 % at 5 years or of 8.6 per 1000 individuals-year (confidence interval (CI) of 95 % 6.9–10.5), being higher in men (10.2, CI 95 % 7.4–13.5) than in women (7.5, CI 95 % 5.5–9.9) (Table 2). The incidence of PAD in subjects < 65 years of age at follow-up was double for men (9.9 per 1000 individuals-year) as compared to women (4.8 per 1000 individuals-year), while in those > 75 years of age, the incidences was similar, at around 12.5 cases per 1000 individuals-year. Overall incidence of PAD was 8.8 per 1000 individuals-year (CI 95 % 6.8–10.8) after direct standardization by the age (>50) and sex European Union structure [25].

**Table 1:** Baseline characteristics of the sample, according to incidence of peripheral artery disease (PAD)

	Without PAD		With PAD		Total		
	*n* = 2161		*n* = 95		*n* ^a^ = 2256		
	n	%	n	%	n	%	*p*
Men	887	41 %	46	48 %	933	41 %	0.153
Age (mean, SD)	63	7.7	66	9.0	63	7.8	0.000
Age category							0.023
49–64	1337	62 %	46	48 %	1383	61 %	
65–69	376	17 %	20	21 %	396	18 %	
70–86	448	21 %	29	31 %	477	21 %	
Education level						0.332	
Illiterate	97	5 %	7	8 %	104	5 %	
Primary education	1482	72 %	63	69 %	1545	72 %	
Secondary education	408	20 %	20	22 %	428	20 %	
University	73	4 %	1	1 %	74	3 %	
Smoking habit							0.000
Never smoker		1285	59 %	37	39 %	1322	58.6 %
Former smoker	532	25 %	31	33 %	563	25.0 %	
Current smoker	344	16 %	27	28 %	371	16.4 %	
Physical activity in leisure time^b^ (mean, SD)	5.8	3.5	4.7	3.7	5.8	3.6	0.009
Physical activity limitations							0.000
None	933	44 %	21	22 %	954	43 %	
Moderate	1052	49 %	56	60 %	1108	50 %	
Can only do light activities	142	7 %	13	14 %	155	7 %	
Any activity causes breathlessness	15	1 %	4	4 %	19	1 %	
Body mass index (Kg/m^2^) (mean, SD)	29	4.5	30	5.6	29	4.6	0.091
Central obesity^c^	1283	60 %	63	66 %	1346	60 %	0.196
Morbidity (clinical record)							
Hypertension	891	42 %	57	62 %	948	43 %	0.000
Hypercholesterolemia	1015	48 %	50	53 %	1065	48 %	0.334
Diabetes	268	12 %	21	22 %	289	13 %	0.006
High LDL (>130 mg/dl)	1225	57 %	53	56 %	1278	57 %	0.783
Low HDL (<40 mg/dl men, <50 mg/dl women)	410	19 %	30	32 %	440	20 %	0.002
High triglycerides (>150 mg/dl)	506	24 %	27	28 %	533	24 %	0.286
Atherogenic dyslipidaemia	199	9 %	11	12 %	210	9 %	0.437
REGICOR score^d^							0.000
≤ 5 %	1253	64 %	32	43 %	1285	64 %	
5–10 %	524	27 %	27	36 %	551	27 %	
> 10 %	166	9 %	16	21 %	182	9 %	
Framingham score^d^							0.000
≤ 10 %	940	48 %	24	32 %	964	48 %	
10–20 %	669	34 %	24	32 %	693	34 %	
> 20 %		334	17 %	27	36 %	361	18 %
Ankle SBP (minimum of both legs) (mmHg) (mean, SD)	147	21	144	24	147	21	0.124
Control brachial SBP (mmHg) (mean, SD)	135	18	142	20	135	18	0.000
Ankle-brachial index (minimum of both legs) (mean, SD)	1.10	0.10	1.01	0.09	1.09	0.10	0.000

*SD* standard deviation, *SBP* systolic blood pressure

^a^Missing values (without PAD + with PAD): education level (101 + 4), physical activity in leisure time (424 + 20), physical activity limitations (19 + 1), body mass index (2 + 0), central obesity (11 + 0), hypertension (28 + 3), hypercholesterolemia (51 + 1), LDL (20 + 0), triglycerides (22 + 0), atherogenic dyslipidaemia (1 + 0), Framingham and REGICOR scores (17 + 0)

^b^Expressed in thousands of METS spent in 14 days doing sporting activities, walking, gardening, walking up stairs, housecleaning and shopping by foot

^c^Central obesity if the circumference of the hip ≥102 (men) or ≥88 (women)

^d^REGICOR and Framingham scores are calculated only in those under 74 years of age at recruitment (*n* = 2035 under 74 years of age)

**Table 2:** Incidence of peripheral artery disease (PAD)

	Overall			Men			Women		
	Cases	I^a^	CI 95 %	Cases	I^a^	CI 95 %	Cases	I^a^	CI 95 %
PAD	95	8.6	6.9–10.5	46	10.2	7.4–13.5	49	7.5	5.5–9.9
Symptomatic PAD^b^	18	1.6	1.0–2.6	11	2.4	1.2–4.3	7	1.1	0.4–2.2
Assymptomatic PAD^b^	71	6.4	5.0–8.1	34	7.5	5.2–10.5	37	5.6	4.0–7.8

^a^Incidence per 1000person-year

^b^2 patients with PAD without information on intermittent claudication and 4 that cannot walk

In healthy patients (normal baseline ABI) a decrease in ABI >10 % was observed in 15 % (*n* = 326), with an increase > 10 % being found in 24 % of the participants (*n* = 510).

In patients presenting a PAD event, the baseline ABI was 1.01, decreasing to 0.80 at follow-up. At follow-up a decrease in ABI >10 % was observed in 83 % of the population with PAD (*n* = 79). The linear correlation between baseline ABI and that at follow-up was low (*r* = 0.23).

Only 1 out of every 4 patients with PAD revealed clinical histories of intermittent claudication, with the remainder being asymptomatic.

The incidence of PAD was significantly associated with male gender, age, smoking habit, major physical activity limitations, arterial hypertension, diabetes and low HDL cholesterol in models adjusted for age, gender and baseline ABI (Table 3).

**Table 3:** Risk factors associated with peripheral artery disease (PAD). Analysis adjusted by age, gender and baseline ankle-brachial index (ABI)

Baseline variables^a^	HR	CI 95 %	*p*
Men	1.81	1.20–2.71	0.011
Age (by year)	1.04	1.01–1.06	0.006
Education level			
Illiterate	1.00		
Primary education	0.71	0.32–1.57	0.288
Secondary education or University	0.85	0.35–2.07	0.385
Smoking habit			
Never smoker	1.00		
Former smoker	2.69	1.45–5.01	0.002
Current smoker	3.78	2.01–7.11	0.000
Physical activity in leisure time^b^ (mean, SD)	0.97	0.90–1.05	0.498
Physical activity limitations			
None	1.00		
Moderate	2.03	1.20–3.42	0.008
Can only do light activities or any activity causes breathlessness	2.55	1.31–4.97	0.006
Body mass index (Kg/m^2^) (mean, SD)	1.03	0.98–1.07	0.234
Central obesity^c^	1.10	0.70–1.73	0.690
Morbidity (clinical record)			
Hypertension	1.71	1.09–2.67	0.018
Hypercholesterolemia	0.98	0.65–1.47	0.905
Diabetes	1.73	1.06–2.84	0.028
High LDL (>130 mg/dl)	0.89	0.59–1.34	0.577
Low HDL (<40 mg/dl men, <50 mg/dl women)	1.82	1.17–2.82	0.008
High triglycerides (>150 mg/dl)	1.05	0.66–1.65	0.846
Atherogenic dyslipidaemia	1.11	0.58–2.12	0.742

^a^Missing values (withoutPAD + with PAD): education level (101 + 4), physical activity in leisure time (424 + 20), physical activity limitations (19 + 1), body mass index (2 + 0), central obesity (11 + 0), hypertension (28 + 3), hypercholesterolemia (51 + 1), LDL (20 + 0), triglycerides (22 + 0), atherogenic dyslipidaemia (1 + 0)

^b^Expressed in thousands of METS spent in 14 days doing sporting activities, walking, gardening, walking up stairs, housecleaning and shopping by foot

^c^Central obesity if the circumference of the hip ≥102 (men) or ≥88 (women)

Using a model adjusting for the baseline ABI, the following variables were significantly associated with the incidence of PAD: Age (hazard ratio (HR) 1.04 per year), smoking (HR 3.49), major physical activity limitation (HR 2.36) and low HDL cholesterol levels (HR 1.68) (Table 4).

**Table 4:** Risk factors associated with peripheral artery disease (PAD). Multivariate analysis^a^

Baseline variables^b^	HR	CI 95 %	*p*
Age (by year)	1.04	1.01–1.07	0.003
Low HDL (<40 mg/dl men, <50 mg/dl women)	1.68	1.08–2.63	0.023
Moderate limitation of physical activities	1.99	1.18–3.37	0.010
Only light activities or any activity causes breathlessness	2.36	1.21–4.63	0.011
Former smoker	2.49	1.53–4.05	0.000
Current smoker	3.49	2.07–5.90	0.000

^a^Also adjusted by baseline ankle-brachial index

^b^20 subjects with missing values excluded

The multivariate model showed the risk factors positively and significantly associated with PAD progression (decrease in ABI > 10 %) to be similar to those associated with the incidence of PAD, with the exception of HDL cholesterol and the appearance of a protective effect for the level of education (Table 5).

**Table 5:** Risk factors associated with a decrease of 10 % of the ankle-brachial index (ABI). Multivariate logistic regression model

Baseline variables^a^	OR	CI 95 %	*p*
Age (by year)	1.03	1.01–1.04	0.001
Education level			
Illiterate	1.00		
Primary education	0.52	0.33–0.83	0.005
Secondary education or University	0.65	0.39–1.08	0.097
Smoking habit			
Never smoker		1.00	
Former smoker	1.42	1.08–1.85	0.011
Current smoker	2.04	1.51–2.75	0.000
Physical activity limitations			
None	1.00		
Moderate	1.34	1.05–1.72	0.018
Only light activities or any activity causes breathlessness	1.22	0.78–1.90	0.388

^a^123 subjects with missing values excluded

## Discussion

To our knowledge, ARTPER is the first population study carried out in Spain to evaluate the incidence of PAD after 5 years of follow-up. The incidence of PAD was 8.6 per 1000 individuals-year, being higher in men than in women, in both the symptomatic and asymptomatic disease.

Previous examinations of incidence carried out in Spain were not population-based studies [13, 14], and obtained incidences between 14.3 (Lahoz) [13] and 23.8 (Merino) [14] per 1000 individuals-year with 4 and 5 years of follow-up respectively. The study conducted by Lahoz et al [13] was carried out on volunteer subjects of both genders (35.9 % male) between 60 and 79 years of age, recruited from one primary health care centre. The greater incidence of PAD found in this study may be explained by the higher age of participants, since PAD increases in both genders with age [4, 14]. The higher incidence found in the study by Merino et al [14] performed in men between the ages of 55 and 74 attending four primary health centres and selected by simple randomization, may be due to the high prevalence of smokers found in this population (48.9 % ex-smokers and 27.8 % active smokers). In addition, the study subjects were exclusively males and patients rather than a general population.

The incidence of PAD in our study was also lower than that found in the majority of European and North American studies [15, 16, 19, 26, 27] which range between five and 44 per 1000 individuals-year.

In the Multi-Ethnic Study of Atherosclerosis (MESA) [15], an incidence of 5.0 per 1000 individuals-year was found in subjects with an average age of 62 years and in which 50 % of the participants were female. In the ARIC study [27], with a follow-up of 10.3 years, the incidence was 13.9 per 1000 individuals-year, probably due to the study being carried out in a diabetic population. In the Cardiovascular Health study [16] the incidence was 15.8 per 1000 individuals-year, which may potentially be explained by the increased age of the participants, almost 10 years older (74.8 ± 4.8) than in our study. However, the highest prevalence (44 per 1000 individuals-year) was found in the BARI 2D study [19] carried out with diabetic subjects with stable coronary heart disease.

On the other hand, the mean ABI of this study (1.099) was similar to that in other studies. Lahoz et al [13] reported a mean ABI of 1.07, similar to that found in the Cardiovascular Health Study [16] despite the participants being older than in our study. In the Edinburg Health Study [26], including subjects between the ages of 55–74, the mean ABI was 1.08. In the MESA study [28, 29] the mean ABI was 1.11, in a population in which the mean age was similar to that of our study (61 vs 63 years of age).

With regard to the decrease in ABI values along the follow up period, a wide variability is described in the different studies published to date probably due to the inclusion of participants with and without PAD. Lahoz et al [13] (750 volunteers) found a decrease in ABI after 4 years of 0.02 ± 0.12 points and in the Edinburg Artery Study [26] (695 participants), there was a decrease in ABI values of 0.01 points across the 12 years of follow-up. The 6-year Cardiovascular Health follow-up study [16] (2071 participants), described differences in ABI in subjects with PAD (decrease of 0.33 ± 0.12) compared to healthy subjects (0.02 ± 0.13), being values similar to those found in our study with a decrease of 0.22 ± 0.13 and an increase of 0.02 ± 0.13, respectively.

### Progression of PAD

The results of studies on the progression of PAD and the criteria used to define progression also vary greatly. In the present study we found that 15.0 % of the subjects showed a decrease in ABI values greater than 10 % at 5 years of follow- up, while Lahoz et al [13] found a decrease in 21.6 % of the volunteer participants. Kallio et al [18] reported progression of PAD (decrease of ABI >15 %) in 19 % of the subjects after 11 years of follow-up. In the Cardiovascular Health Study [16], PAD progression was found (decrease of ABI > 15 % or ABI <0.9) in 9.5 % of the subjects after 6 years of follow-up, being associated with an increase in morbidity-mortality.

### Variables associated with peripheral artery disease incidence

The risk factors associated with the incidence of PAF in this study were age, male gender, arterial hypertension, diabetes and low HDL cholesterol and their presence almost doubles the risk of presenting PAD during follow-up Major physical activity limitations and active smoking also increased the risk of PAD incidence by 2.5 and 4 times, respectively.

However, obesity, central obesity, hypercholesterolemia, high LDL cholesterol and hypertriglyceridemia were not found to be associated with the incidence of PAD similar to what has been suggested by other authors [13, 14].

Age is a risk factor associated with the progression of PAD in most of the studies published to date [10, 15, 16, 30]. This is probably due to the increase in the prevalence of different risk factors with age, thereby explaining the disappearance of these factors disappear in the multivariate model adjusted for age. The progression of arteriosclerosis also advances with age, likely because, as some authors suggest, of the longer exposure to risk factors over time [31]. However, in our study we found that in participants < 65 years of age, the incidence of PAD in men is practically double that of women. On the other hand, in subjects over the age of 75 years, the incidence of PAD is much higher, albeit more equal for both genders.

As in the majority of studies, we found an association between male gender [13, 15, 19] and the incidence or the progression of PAD. This may be explained by the increased prevalence of smoking in males or, as suggested by some authors, due to genetic predisposition. However, paradoxically, in some studies fewer cardiovascular risk factors as well as a lower incidence of PAD events and, contrarily, an increased predisposition for PAD were found in women [32].

In general, as described in the literature and as found in the present study smoking, arterial hypertension and diabetes are the most important risk factors associated with the incidence and progression of PAD [14, 15, 27]. Different studies, including ours, have also found an inverse association with HDL cholesterol figures [33]. In our study, only age, smoking, HDL cholesterol and a less-studied variable, decreased physical activity, remained in the multivariate model. On the other hand, despite showing a relationship with PAD in the non- adjusted models, factors such as hypertension or diabetes were not included in the multivariate model. The relatively low number of cases studied and the correlation with other variables may explain why these variables were not associated with PAD in the multivariate model

### Strengths and limitations of the study

The strengths of this study include the fact that it was carried out in the general population, used follow-up via telephone and in-person in the second ABI measurement, was carried out by trained individuals, used standardized measures for all participants, confirmed clinical risk factors based on clinical history, had high participation during the 5 years of follow-up and performed quality control of the data analyzed, all of which increase the validity of the results obtained.

However, although participation at 5 years was high (77 %), significant differences were found between the participating and non-participating patients, as found by other authors [1]. Older subjects and those with more pathologies were those that did not participate in the second ABI measurement which may lead to an underestimation of incidence in our study, and may alter the presence of other risk factors associated with the appearance of PAD. However, this limitation is inherent in most longitudinal studies, particularly in those following older individuals. Our results do not differ greatly from those published previously; however it does provide more valid information, in terms of the sample studied, and with results from Spain.

The limited correlation found between the results of the baseline ABI and those of the follow- up (*r* = 0.23) is another potential limitation of the study, perhaps because the ABI was based on a single determination taken at one specific time. Moreover, inter and intra observer variability could affect this correlation. This may decrease the reliability of the results, although it is also another common limitation in all studies. To improve this, it may be more appropriate to determine the ABI on three occasions, separated by a reasonable amount of time and obtaining a result being the mean of the 3 determinations, as done for the diagnosis of arterial hypertension.

The incidence of PAD, based on an ABI in the second determination was calculated at the same time that the measurement was performed. This may lead to an artificial extension of observation time for the participants with PAD events, since the event probably occurred at some point between recruitment and the second ABI measurement. This may also lead to a slight underestimation of the incidence of PAD, since the time of diagnosis is delayed. In any case, this is inevitable and occurs in most studies on incidence, in which the time disease diagnosis does not coincide with the actual appearance of the disease in the patient.

## Conclusions

The incidence of PAD in the ARTPER cohort is 8.6 cases per 1000 individuals-year, being higher in men than in women, particularly in those under the age of 65. Smoking, age and physical activity limitations are the factors that are the most frequently associated with a decrease in ABI and the appearance of PAD. The correlation between 2 ABI measurements, separated by 5 years of follow-up is low; therefore it may be necessary to examine whether repeated measures of the ABI might improve this correlation.

## Abbreviations

ABI: Ankle-brachial index
PAD: Peripheral arterial disease
